# Feedback systems as interferers in perfectionism: a systematic literature review

**DOI:** 10.3389/fpsyt.2025.1732312

**Published:** 2026-01-14

**Authors:** Adel Bartos, Monika Marosi, Istvan Karsai, Kristof Schwartz

**Affiliations:** 1 Doctoral School of Education, ELTE Eötvös Loránd University, Budapest, Hungary; 2 Institute of Health Promotion and Sport Sciences, ELTE Eötvös Loránd University, Budapest, Hungary; 3 Doctoral School of Regional- and Business Administration Sciences, Széchenyi István University, Győr, Hungary; 4 Sports and Physical Education Center, Medical School, University of Pécs, Pécs, Hungary; 5 Department of Psychology and Health Management, Faculty of Health and Sports Sciences, Széchenyi István University, Győr, Hungary

**Keywords:** emotional regulation, feedback, perfectionism, personality traits, psychophysiological response

## Abstract

**Background:**

Perfectionism is a multidimensional construct characterized by the striving for exceptionally high standards and critical self-evaluation. It can manifest in both adaptive and maladaptive forms. Feedback systems exert a considerable cognitive influence on individuals as the emotional and behavioral responses to feedback are often shaped by its valence—positive or negative. This study aimed to examine the relationship between feedback systems and perfectionism, including its various dimensions, and to assess how specific interventions influence perfectionistic traits.

**Methods:**

A systematic literature review was conducted following the PRISMA (Preferred Reporting Items for Systematic Reviews and Meta-Analyses) guidelines across six academic databases: PubMed, PsycINFO, Scopus, ScienceDirect, EBSCO, and ERIC. The initial search yielded 441 articles. After applying the inclusion and exclusion criteria, 24 studies were selected for detailed analysis.

**Results:**

A clear association emerged between feedback valence and perfectionism. Four major outcome domains were identified as dependent variables: emotional response, behavior, task performance, and physiological (biomarker) indicators. Among adaptive perfectionists, positive feedback was linked to improved behavioral outcomes, whereas negative feedback elicited negative emotional and performance-related consequences. In contrast, maladaptive perfectionists showed a heightened vulnerability to negative feedback, displaying impaired emotional regulation, decreased performance, and elevated stress-related physiological markers.

**Conclusion:**

Feedback directed at individuals with perfectionistic traits elicits distinct psychological and physiological responses. While positive feedback can foster beneficial outcomes in adaptive perfectionists, negative feedback—especially in maladaptive perfectionists—can have substantial adverse effects, highlighting the importance of developing individualized feedback strategies as part of the clinical and therapeutic interventions for individuals with perfectionistic vulnerability.

**Systematic review registration:**

https://www.crd.york.ac.uk/prospero/, identifier CRD420251015998.

## 1 Introduction

Feedback, as a component of social interaction, is a term universally used in the field of psychology (1). The study of feedback and its developmental relevance is reflected in a number of disciplines, ranging from child education (2), public education (3), art (4), and sports activity (5) to organizational behavior management (6), resulting in interdisciplinary research findings applying the science of communication and psychology. Due to the diversity of the listed disciplines, it is necessary to use a standardized version of stakeholders appearing in the current study so that the collective term “teacher, instructor, coach, leader” will be referred to as power-holder and, in addition, “pupil, student, athlete” will be referred to as power-receiver. Feedback is a source of information based on observation, which is essential for mastery of the performed tasks. It is generated between members of the asymmetric dyad (subordinate and superior participants, e.g., in a teacher–student relation) and is delivered by the power-holder during or immediately after the activity is accomplished (6). Appropriate feedback is carefully worded, mainly consisting of verbs and nouns; furthermore, it is also constructive, descriptive, and non-judgmental information (7). The message, expressed in an optimal tone of voice, non-threatening and without sarcasm, creates a positive communication environment for the recipient, thus providing an opportunity to improve performance (7). The types of feedback have been categorized in diverse ways (8). Highlighting a threefold division, feedback with a high level of informational content is considered the most effective (9). In this case, the power-holder provides information not only about the outcome of the task but also about how the power-receiver performed the task. The second type under this classification is corrective feedback, in which, compared with the previous one, the power-holder only informs about the correctness or incorrectness of the result, possibly accompanied by the solution to be corrected. As a third type, in a lack of providing improvement and corrective information, the power-holder rather enacts the positive or the negative consequence of the task performed—this is the reinforcing or punitive form of feedback (8). According to the evaluation of university students, learner-centered feedback—which is most closely aligned with high information-level feedback from the above list—tends to be more effective than traditional methods and is also more positively rated if not provided in the form of value judgement at the end of the task but during its implementation, providing an opportunity for a dialogue (10).

Feedback systems have significant cognitive impacts on the human body (3). Feedbacks can enhance self-confidence (11) as they can activate the reward system of the brain, including dopaminergic pathways. Positive and negative feedbacks are processed by separate neural networks, and oscillations of varied frequencies indicate different dopaminergic signaling transduction (12). Some studies suggest that feedback can improve the learning strategies and knowledge acquirement (13), but does not affect the motivation during learning (14), underscoring that some findings only claim a moderate effect on learning (3). Feedback can reinforce correct decision making and enhance memory retention (15).

However, negative feedback can exert a dual effect: it can encourage development and performance improvement constructively (16) or it can reduce self-confidence and distort self-image destructively (17). Because feedback is one of the most important instruments of learning due to neuroplasticity, biofeedback is gaining scientific and market interest as a therapeutic application that can help regulate neurological and psychological processes (18). While feedback may even be used for therapeutic purposes, some scholars have highlighted its stress-enhancing factor (19).

Perfectionism was initially identified as a human quality that implies the need to achieve the best possible performance and the related critical view (20). Later, it was interpreted more as a characteristic that is generally manifested in the behavior of individuals (21). Nevertheless, currently, the term disposition is more commonly used (22). The antecedents of perfectionism development and the consequences of its existence have been summarized in six dimensions: concerns about mistakes, personal expectations, doubts about action, parental expectations, parental criticism, and organization (20). In this case, the hypothesized antecedent is represented by two dimensions related to parents, while others characterize the perfectionist’s behavior and conceptual world. In contrast to the six-dimensional model focusing on self, the three-dimensional model (23), emphasizing the social environment, does not aim antecedent and consequent mapping, but rather reveals direction along the following divisions: perfectionism toward oneself, perfectionism toward others, and socially or socially imposed perfectionism, thus perfectionism of external expectations. Beyond the aforementioned two main models, other alternative approaches have also been adopted, with their associated measurement frameworks. Previously understood as a one-component, exclusively negative phenomenon (24), perfectionism is treated by the current approaches as a multidimensional construct (20, 23), distinguishing between the adaptive and maladaptive forms (25). Adaptive perfectionism involves a goal-oriented focus on performance and striving for positive outcomes, while the maladaptive form is associated with excessive self-criticism, anxiety, and failure-avoidant behavior (26). The development of perfectionism and its consequential constituents appear to be referred to by most researchers as traits built through social learning and experience acquired from the environment (27), while the possibility of genetic predisposition cannot be excluded either (28). Perfectionism is a widely researched phenomenon not only in the fields of education and sports science but also in medicine, offering an interdisciplinary research topic.

In the first multidimensional models of perfectionism (20), parental expectations and parental criticism already appear as distinct factors, behind which the feedback patterns regularly experienced by the child can be assumed. This suggests that feedback is not merely an external environmental influence but also a theoretically integrated component of the development of perfectionism. Approaches based on social learning and the internalization of interpersonal expectations (27, 29, 30) further indicate that the formation of personal standards and self-critical evaluative patterns may also be partly linked to the feedback processes received from parents, teachers, or other individuals in positions of authority. This implies that, although direct empirical evidence with regard to the developmental origins of perfectionism is limited, theoretical models nonetheless outline a possible pathway through which feedback systems may contribute to the influencing perfectionistic functioning. However, there is currently no unified and coherent standpoint in the literature that clearly and consistently explains how feedback relates to perfectionism. To our knowledge, no systematic review has yet been conducted on the topic.

In this study, we seek to answer the question of the relationship between feedback systems and certain characteristics of perfectionism through a systematic review. The question arises whether there is a link between maladaptive perfectionism resulting from certain types of feedback systems and whether feedback systems possess such a profound personality-forming power.

## 2 Methods

Emerging from the complexity and the aim to understand the interdisciplinarity of the topic, the basis of our research lies on a systematic literature review (SLR) that unites analysis of relevant scientific research from 1995 onwards. The PRISMA (Preferred Reporting Items for Systematic Reviews and Meta-Analyses) (31) protocol constitutes the main framework of the review, under which available empirical publications on the roles of instructions and feedback as psychological affects have been accentuated. Our analysis consisted of the following steps.

### 2.1 Collection of literature sources

The literature was searched using PubMed, PsycINFO, Scopus, Science Direct, EBSCO, and ERIC scientific databases using the following keywords: “perfectionism,” “feedback,” “instruction,” and their combinations. The date of publication for the literature sources was not specified under the search criteria. The source collection and SLR were conducted in October 2025 through the search protocol and study in accordance with the registration requirements of PROSPERO, under registration no. CRD420251015998. The selection of articles and the extraction of data followed a structured multistep procedure. The screening of studies and the extraction of data were conducted by two independent reviewers. Decision making followed a predefined procedure consisting of three steps: 1) each reviewer independently assessed the studies based on the established inclusion and exclusion criteria; 2) in cases of disagreement, a bilateral discussion was held to clarify the basis of the decision; and 3) if consensus could not be reached through discussion, a third expert was involved to adjudicate the final decision. An inter-rater reliability was not formally calculated as the decisions required qualitative judgment, and the heterogeneity and multidimensional nature of the included studies—addressing the interplay of intervention methods, feedback processes, and perfectionism—did not allow for consistent statistical coding. Reliability was ensured through independent assessment, consensus-based resolution, and, when necessary, the involvement of a third expert.

### 2.2 Inclusion and exclusion criteria

The inclusion criteria indicated that articles had been published in peer-reviewed journals and in English, containing a combination of the aforementioned keywords, in addition to discussing the relationship between feedback systems and perfectionism. As a further criterion, studies in which intervention trials were employed were selected.

Therefore, publications which fell under type segmentation of conference papers and volumes, theses, dissertations, chapters, and books; were not written in English; and did not involve any combination of the searched keywords nor were closely related to the issue of perfectionism and feedback systems in their entirety were excluded. Finally, based on the exclusion criteria, descriptive literature reviews, meta-analyses, or studies based solely on cross-sectional examinations without intervention were also removed. The focus on intervention studies was a deliberate methodological choice. Previous systematic reviews in the field of perfectionism have predominantly identified cross-sectional research and have consistently noted this as a limitation (32–34), while the broader literature has also highlighted the scarcity of longitudinal studies (27). In light of these considerations, the present review concentrated on intervention-based research that empirically examined the relationship between feedback systems and perfectionism. Accordingly, the search strategy employed keywords specifically related to feedback processes. Terms such as “parental expectations” and “parental criticism,” although closely linked to the dimensions of perfectionism, do not represent discrete feedback constructs and were therefore not suitable as specific search criteria for the scope of this review.

### 2.3 Data extraction and quality considerations

The inclusion of heterogeneous perfectionism measures reflects the theoretical diversity of the field, as no single definition or measurement approach has achieved consensus. Accordingly, previous systematic reviews have likewise integrated studies that employed multiple validated instruments (32–35), and the present review followed this established practice as limiting the synthesis to a single measure would substantially narrow the available empirical evidence.

A formal risk-of-bias or study quality assessment tool was not applied due to the heterogeneity of the study designs and the outcome measures across the included interventions, which precluded the use of a single standardized framework. Instead, the methodological strengths and weaknesses were evaluated narratively, and the implications of this approach are addressed in *Section 4.5*.

## 3 Results

**Figure 1:**
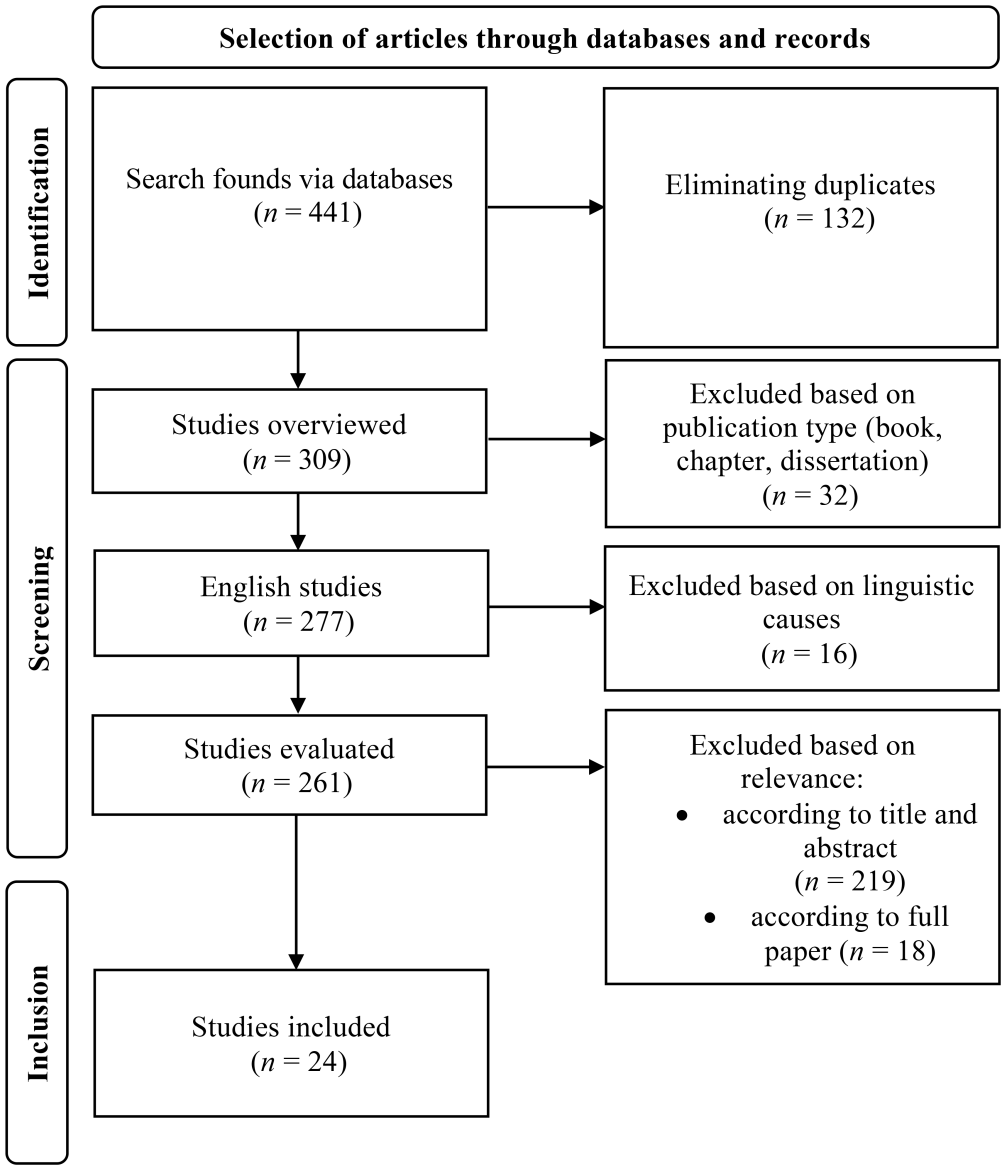
Flowchart of the systematic literature review based on PRISMA (Preferred Reporting Items for Systematic Reviews and Meta-Analyses). Adapted from Page et al. (31), PRISMA 2020 flow diagram.

Using the aforementioned search engines, 441 articles including the above-mentioned keywords as the terms of search were found. After eliminating duplicates and then applying the selection criteria, 24 articles were finally selected, the selection process of which is shown in **Figure 1**.

In finalizing the literature screening, only two studies were excluded due to their cross-sectional design in terms of research methodology, in contrast to all the other 24 studies that employed intervention trials.

Accordingly, the results table (**Table 1**) shows that the feedback systems we searched for appear as the main elements of intervention, occurring as independent variables. The other element under observation, perfectionism, does not emerge as a typical dependent variable in the studies examined. Part of our problem statement suggesting that feedback may have a potential personality-shaping power to support the development of perfectionism remains unclear as, in the analyzed studies, perfectionism is presented as already developed, constituting the main pillar of the research. Hence, our examination focus is on whether feedback received as an intervention has an impact on the cognitive functions, feelings, behavior, and, where appropriate, the performance of individuals with perfectionism. Another aspect of our problem statement—i.e., whether there is a relationship between perfectionism, its dimensions, and feedback systems—is comprehensively outlined in the paper through a table of results.

**Table 1:** Summary of the studies selected.

No.	Study	Measuring instrument for perfectionism	Intervention	Measuring instrument for further variables	Subjects examined	Results
1	Hobden and Pliner (1995) (48)	MPS	Intelligence test Default positive feedback in verbal form	Choice to influence performance	Total = 84 (F = 42; M = 42) Nationality: Canadian	Those with high SPP exhibited significantly higher levels of self-handicapping, but only in public settings. In private conditions, a significant relation was not observed. High levels of SOP were associated with significantly greater self-handicapping in all settings compared with those with low SOP, whether private or public.
2	Besser et al. (2004) (41)	MPS	Choice reaction time response Written negative or positive feedback in random grouping	Visual Analogue Scale (VAS) Cognitive and performance appraisals Objective performance	Total = 200 (F = 100; M = 100) *M*_age_ = 21.75 years; SD = 3.08 Nationality: Israeli	Following positive feedback, those with high SOP showed a significant increase in positive emotions compared with those with low SOP; however, after negative feedback, they experienced fewer positive emotions. Those with high SOP showed a significant increase in their negative emotional state after both positive and negative feedback. No relationship was found between SPP and a significant increase in negative emotions, nor a significant decrease in positive emotions following post-task feedback. After negative feedback, those scoring high at SOP were significantly more ruminative than those with low SOP. In addition, they exhibited significantly greater rumination when they received negative feedback than when receiving positive feedback.
3	Anshel and Mansuori (2005) (50)	FMPS	Total body-balancing task False verbal negative feedback	Children’s Arousal Scale (CAS-A)	Total = 30 (F = 0; M = 30) Age = 19.6–22.8 years Nationality: American	Those with higher perfectionism scores presented a significantly lower performance in the balancing task due to negative feedback.
4	Besser et al. (2008) (36)	MPS PCI	Choice reaction time response Written negative or positive feedback in random grouping	Visual Analogue Scale (VAS) State Self-esteem State Automatic Thoughts Questionnaire (ATQ and ATQ-P) Heart rate Systolic blood pressure	Total = 200 (F = 100; M = 100) *M*_age_ = 23.63 years; SD = 2.92 Nationality: Israeli	As a result of positive feedback, both those with high and low SPP displayed an increase in positive emotion. On the other hand, a decrease in positive emotions was more likely among those with high SPP when receiving negative feedback. Increased anxiety levels were observed in the case of negative feedback when a high SPP was combined with a high self-confidence and in the case of positive feedback when a high SPP was combined with a low self-confidence. SPP can be associated with low post-task performance self-evaluation, which was significantly stronger due to negative feedback. A high SPP in combination with a low self-confidence resulted in an increased heart rate after negative feedback, but was augmented significantly less due to positive feedback. A high SPP was significantly linked to an increase in systolic blood pressure as a result of negative feedback, but no such pattern was found for positive feedback.
5	Stoeber et al. (2008) (39)	Striving for Perfection Scale	Raven’s Advanced Progressive Matrices Verbal negative or positive feedback in random grouping	Self-criticism Subscale of the Revised Attitudes Toward Self-Scale General Self-Efficacy Scale Aspiration level	Total = 100 (F = 82; M = 18) *M*_age_ = 21.2 years; SD = 6.7 *n*_failure_ = 50; *n*_success_ = 50 Nationality: English	After negative feedback, the self-confidence of highly self-critical individuals decreased significantly more compared with those with low self-criticism; however, no significant relationship was found due to positive feedback. The aspiration levels of those with high perfectionist aspiration showed a significantly greater increase compared with those with low aspiration, but only after positive feedback, with no detectable relationship found as a result of negative feedback.
6	Kobori et al. (2009) (45)	MPS	Modified Stroop Task default positive feedback	General Affect Scale	Total = 53 (F = 27; M = 26) *M*_age_ = 22.00 years; SD = 1.5 Nationality: Japanese	Subjects with high SOP scores significantly choose the harder goal over the easier one before the next task.
7	Diamond et al. (2012) (46)	MPS	Computer game task Verbal negative or positive feedback in random grouping	Center for Epidemiological Studies—Depression Scale (CES-D) Goal setting behavior	Total = 95 (F = 95; M = 0) Age = 22–24 years; SD = 1.1 *n*_failure_ = 48; *n*_success_ = 47 Nationality: Israeli	The target setting of those with low SOP and high SPP increased largely when they received positive feedback, but decreased when receiving negative feedback.
8	Egan et al. (2012) (47)	FMPS CPQ	Nonverbal logic test Negative or positive feedback	The Positive and Negative Affect Schedule (PANAS) Visual Analogue Scales (VAS)	Total = 206 (F = 152; M = 54) M_age_ = 30.56 years; SD = 13.25 Nationality: Australian	The PS_2_ score was significantly correlated with the target set for each task regardless of feedback. The CPQ showed no significant relationship with serial goal setting, but with feedback on success or failure influenced further goal setting.
9	Azam et al. (2015) (55)	MPS PCI	Unsolvable pattern recognition task Written negative or positive feedback in random order	HRV	Total = 60 (F = 29; M = 31) *n*_maladaptive_ = 21; *M*_age_ = 22.05 years; SD = 2.57 *n*_not perfectionists_ = 39; *M*_age_ = 20.62 years; SD = 0.82 Nationality: nk	Perfectionists did not show a significantly lower HRV in response to stress compared with the control group. In contrast to the control group, perfectionists did not show a significant decrease in HRV compared with the baseline under stress.
10	Cotet and Veresezan (2015) (44)	MPS	Cognitive task Default negative feedback	The General Attitudes and Beliefs Scale—Short Form (GABS-SF) The Generalized Self-Efficacy Scale Measuring negative functional and dysfunctional affective forecast Measuring perceived task difficulty	Total = 95 (F = 84; M = 11) *M*_age_ = 20.33 years; SD = 2.27 Nationality: nk	Both high SOP and high SPP revealed a significant relationship with the absolute accuracy of anger prediction; thus, prediction of anger was more inaccurate for perfectionists.
11	Muralidharan et al. (2015) (42)	DAS—Perfectionism subscale	Affective Stroop Task Default negative feedback in written form	Depressive Experiences Questionnaire (DEQ) Perceived Criticism (PC) Parental Bonding Instrument (PBI) Positive and Negative Affect Schedule (PANAS) Five-Minute Speech Sample (FMSS) Hamilton Depression Rating Scale (HDRS) Young Mania Rating Scale (YMRS) Interviews	Total = 44 (F = 20; M = 24) *n*_bipolar_ = 22 *M*_age_ = 25.18 years; SD = 4.05 *n*_control_ = 22 *M*_age_ = 26.44 years; SD = 5.44 Nationality: American	Affective reactivity to negative feedback showed no significant relationship with cognitive schemas of self-criticism and perfectionism.
12	Geisler et al. (2017) (56)	EDI-2—Perfectionism subscale	Probabilistic reversal learning task Negative or positive written feedback	IQ—Wechsler Adult Intelligence Scale (WIE)/Wechsler Intelligence Scale for Children (HAWIK) Beck Depression Inventory (BDI-II) Body Mass Index (BMI) fMRI	Total = 72 (F = 72; M = 0) *n*_anorexia nervosa_ = 36; *M*_age_ = 16.0 years; SD = 2.6 *n*_control_ = 36; *M*_age_ = 16.3 years; SD = 2.6 Nationality: nk	In the AN group, negative feedback was accompanied by an increased dACC response, which correlated with perfectionism, whereas no such relationship was found in the control group.
13	Curran and Hill (2018) (43)	MPS	Cycle ergometer task False written negative feedback	State Shame and Guilt Scale (SSGS)	Total = 60 (F = 7; M = 53) *M*_age_ = 20.78 years; SD = 3.57 Nationality: English	High levels of SOP and SPP indicated a strongly significant forecast of increased shame and guilt as a result of negative feedback following a series of competitive tasks.
14	Lo and Abbott (2019) (37)	APS-R	Mental rotation task Written and verbal negative or positive feedback in random grouping	Positive and Negative Affect Schedule (PANAS) State Anxiety Rating (SAR) Self-Rating Questionnaire	Total = 175 (F = 127;M = 48) *M*_age_ = 19.05 years; SD = 2.26 *n*_failure_ = 88; *n*_success_ = 87 Nationality: nk	Maladaptive perfectionism predicted an increase in negative emotions resulting from initial negative feedback, with strong significance. An increase in positive emotions responding to initial negative feedback was also significantly predicted by maladaptive perfectionism, but positive emotions were significantly reduced with repeated negative feedback. Maladaptive perfectionism predicted self-evaluation of positive traits after an initial positive feedback, with strong significance, and adaptive perfectionism, with significance.
15	Cooks and Ciesla (2019) (38)	MPS FMPS	Anagram and matrix reasoning tasks Negative or positive feedback in random grouping	Positive and Negative Affect Schedule (PANAS) Center for Epidemiological Studies—Depression Scale (CES-D) Psychiatric Epidemiology Research Interview Life Events Scale (PERI)	Total = 125 (F = 92; M = 33) *M*_age_ = 19 years; SD = 1.94 Nationality: nk	Perfectionist concerns predicted an increase in negative affect following negative feedback, whereas no significant relationship was found following positive feedback. During the follow-up stage of the study, a significant relationship was found between perfectionism concerns and high life stress, predicting the improvement of depressive symptoms.
16	Lizmore et al. (2019) (51)	Sport-MPS-2 MIPS	Golf-putting task False negative feedback	Mental Readiness Form (MRF)	Total = 99 (F = 52; M = 47) *M*_age_ = 20.51 years; SD = 1.79 Nationality: Canadian	Following negative feedback, PS_1_ was associated with better performance if the PC is lower, but worse performance if the PC is higher.
17	Luna et al. (2021) (57)	IPI	Education of the Polish ringo game MooN program based on the Sport Education Model (SEM)	The Self-Efficacy Inventory for Multiple Intelligences (IAMI)	Total = 170 (F = 97; M = 73) *M*_age_ = 10.76 years; SD = 0.73 Nationality: Spanish	Implementing the MooN program, ANOVA revealed a small effect, but a significant difference for self-reported perfectionism, although the mean value of the Perfectionism Scale did not show significant difference in the experimental group compared with the control group.
18	Rahmaty and Zarei (2021) (58)	MPS	Foreign language education Dynamic Assessment (DA)	Foreign Language Classroom Anxiety Scale (FLCAS) “Willingness to Communicate Inside the Classroom” questionnaire	Total = 166 (F = 146; M = 20) *M*_age_ = 15 years; SD = 2.4 Nationality: Iranian	No significant differences between the application of interactionist dynamic assessment, interventionist dynamic assessment, and the traditional education method were stated when exerting influence of perfectionism among students learning English as a foreign language.
19	De Muynck et al. (2021) (40)	Self-Critical Perfectionism (subscales from the FMPS)	Series of tennis exercises Negative or positive normative feedback in random grouping	Perceived Competence Scale Basic Psychological Need Satisfaction and Frustration Scale Flow State Scale Experienced tension	Total = 59 (F = 18; M = 41) *M*_age_ = 15.47 years; SD = 1.63 Nationality: Belgian	Self-critical perfectionism showed a significant inverse relationship with current satisfaction of competence need, which was enhanced by negative feedback.
20	Isheqlou et al. (2022) (53)	APS-R	Monetary gambling task Written negative or positive feedback		Total = 60 (F = 42; M = 18) Age = 18–30 years *n*_adaptive_ = 22 *n*_maladaptive_ = 20 *n*_non-perfectionists_ = 18 Nationality: nk	Maladaptive perfectionists had faster reaction times than members of the adaptive and non-perfectionist groups, with strong statistical significance, regardless of positive or negative feedback. Both adaptive and maladaptive perfectionists presented the fastest reaction times following negative feedback.
21	Dunkley et al. (2023) (59)	FMPS-SF HFMPS-SF APS-R-SF DEQ-SF	Personalized feedback (for perfectionists)	Mood and Anxiety Symptom Questionnaire Short Form (MASQ) Patient Empowerment Scale Dispositional Version of the COPE Inventory Beck Depression Inventory (BDI) Coping Self-Efficacy Scale (CSES)	Total = 176 (F = 148; M = 28) *M*_age_ = 21.39 years; SD = 2.72 nationality: nk	As a result of the intervention, the self-affirmation, coping self-efficacy, and problem-focused coping of perfectionists in the intervention group significantly increased compared with the control group. The intervention resulted in a significant reduction of the depressive and anxiety symptoms among perfectionists in the intervention group compared with those in the control group.
22	Isheqlou et al. (2023) (54)	APS-R	Monetary gambling task Written negative or positive feedback	Beck Anxiety Inventory (BAI) EEG/ERP	Total = 58 (F = 40; M = 18) *n*_adaptive_ = 19; *M*_age_ = 22.26 years; SD = 3.1 *n*_maladaptive_ = 21; *M*_age_ = 21.95 years; SD = 2.48 *n*_non-perfectionists_ = 18 *M*_age_ = 20.94 years; SD = 1.73 nationality: Iranian	Adaptive perfectionists had significantly longer response times to tasks than maladaptive perfectionists and non-perfectionists. The FRN amplitude was observed to be significantly higher in response to negative feedback among adaptive perfectionists compared with maladaptive perfectionists.
23	Portešová et al. (2023) (49)	FMPS	Logic video game (Triton) Feedback in points	Cattell’s Fluid Intelligence Test (CFT 20-R) Big Five Inventory-2-Short Form (BFI-2-S)	Total = 133 (F = 62; M = 71) *M*_age_ = 12.4 years; SD = 0.40 Nationality: Czech	Functional perfectionists used point monitoring representing feedback significantly more often during the game compared with dysfunctional perfectionists. Task manipulation as a competitive condition resulted in significantly greater increases in the GPMF for functional perfectionists than for dysfunctional perfectionists.
24	Zhou et al. (2025) (52)	Perfectionism Scale	Cognitive task False negative feedback (self-comparison or social comparison)	Executive function	Total = 75 *M*_age_ = 19.49 years; SD = 1.08 Nationality: Chinese	Only the group with high PS1 and PC (mixed perfectionists) showed significant differences in reaction time, with shorter reaction times following social-comparative negative feedback than after self-comparative negative feedback.

*MPS (HF-MPS)*, Hewitt–Flett Multidimensional Perfectionism Scale; *HFMPS-SF*, Multidimensional Perfectionism Scale—Short Form; *FMPS*, Frost Multidimensional Perfectionism Scale; *FMPS-SF*, Multidimensional Perfectionism Scale—Short Form; *APS-R*, Almost Perfect Scale—Revised; *APS-R-SF*, Almost Perfect Scale—Revised—Short Form; *CPQ*, Clinical Perfectionism Questionnaire; *DEQ-SF*, Depressive Experiences Questionnaire—Short Form; *PCI*, Perfectionism Cognitions Inventory; *IPI*, Childhood Perfectionism Inventory; *EDI-2*, Eating Disorders Inventory; *DAS*, Dysfunctional Attitude Scale—Form A; *Sport-MPS-2*, Sport Multidimensional Perfectionism Scale 2; *MIPS*, Multidimensional Inventory of Perfectionism in Sport; *SOP*, self-oriented perfectionism; *SPP*, socially prescribed perfectionism; *PS_1_*, perfectionistic strivings; *PS_2_*, personal standards; *PC*, perfectionistic concerns; *EFI*, explanatory feedback intervention; *EEG*, electroencephalogram; *FRN*, feedback-related negativity; *HRV*, heart rate variability; *AN*, anorexia nervosa; *dACC*, dorsal anterior cingulate cortex; *GPMF*, game points monitoring frequency.

*p* ≤ 0.01 (strong significance); *p* ≤ 0.05 (significance).

## 4 Discussion

The primary aim of the current study was to explore whether there is a link between feedback systems and perfectionism. In addition, we sought to answer the question of what, if any, direction such a relationship might take and to what extent and in what ways the feedback types might influence the adaptive and maladaptive forms of multidimensional perfectionism.

### 4.1 Classification by dependent variables related to emotions

Emotional and cognitive reactions to negative feedback obtained more prominent emphasis in the studies. In response to negative feedback, maladaptive perfectionists showed a decrease in positive emotions (36, 37), an increase in negative emotions (37, 38), an elevated anxiety level (36), and a low self-esteem (36, 39). On the other hand, an inverse significant relationship with competence satisfaction was also reported (40). Furthermore, a significant relationship was found between perfectionist concerns and high life stress during post-examination follow-up, predicting an increase in depressive symptoms (38). On the contrary, some studies revealed no significant relationship between maladaptive perfectionism and affective reactivity related to negative feedback (41, 42). After similar negative feedback, those with adaptive perfectionism showed a decreased positive affect, an increased negative affect, and rumination (41). Individuals who scored high on both self-oriented perfectionism (SOP) and socially prescribed perfectionism (SPP) demonstrated strong significant predictors of increased shame and guilt affected by negative feedback following a series of competitive tasks (43), and a further significant relationship was detected with the absolute accuracy of predicting anger (44), suggesting that individuals with lower perfectionism are more accurate in predicting anger following failure.

In the positive feedback setting, both adaptive (41) and maladaptive (36) perfectionism maintained significant relationships with increased positive emotions and predicted self-evaluation of positive traits (37), whereas in maladaptive perfectionism combined with low self-confidence, the anxiety level increased as a consequence of positive feedback (36). Finally, several studies detected no traceable relationship with positive feedback on maladaptive perfectionism (38, 39). These patterns indicate that negative feedback may reliably elicit more detrimental emotional outcomes because it preferentially activates the psychological processes associated with a heightened threat appraisal and sensitivity to social evaluation. Beyond perfectionism itself, co-occurring mechanisms—such as a tendency toward self-critical cognitive styles, a reduced perceived control, or an elevated vulnerability to shame—can further intensify the negative affective responses. Such processes may help explain why negative feedback produces more consistent and severe emotional consequences across studies, irrespective of variations in the perfectionism profiles.

### 4.2 Classification by dependent variables related to behavior/action

Multiple studies converged on similar outcomes showing that individuals with high SOP scores tended to choose the harder task at significantly greater frequency following positive feedback (39, 45), which also pertained to those with low SOP but high SPP scores (46). After negative feedback, the set goal was lowered (46), but no detectable correlation was found in this relation (39). The scores in the personal standards (PS) dimension generally correlated with goal settings, regardless of feedback, while the Clinical Perfectionism Questionnaire (CPQ) showed no significant relationship (47). Following positive feedback, participants with high SPP exhibited significantly higher levels of self-doubt, but only in public settings; in private, this pattern was not observed. In contrast, high levels of SOP were associated with significantly greater self-handicapping in all settings, whether private or public (48). Similarly indicating behavioral patterns in another study, functional perfectionists used point tracking representing feedback significantly more often during the game compared with dysfunctional perfectionists. In addition, the task condition manipulated in a competition context resulted in a stronger increase in the point monitoring frequency for functional perfectionists (49). It appears that positive feedback plays a salient role in the behavioral responses to different aspects of perfectionism.

### 4.3 Classification by dependent variables related to performance

As a consequence of negative feedback, subjects scoring higher at perfectionism performed considerably worse in the balancing tasks (50). Similarly, those with a combination of high perfectionistic strivings (PS) and perfectionistic concerns (PC) exhibited inferior performance on the golf task (51). Only those participants with high PS (perfectionistic strivings) and PC showed remarkable differences in reaction time: they showed faster reaction times after peer-comparative negative feedback than after self-comparative negative feedback (52). The fastest reaction time in the cognitive task performance among both adaptive and maladaptive perfectionists was also associated with negative feedback (53), although maladaptive perfectionists had faster reaction times than those in the adaptive and non-adaptive groups, demonstrably through strong significance and regardless of the direction of feedback. In absolute accordance with the previous result, and also independent of the feedback direction, the response times of adaptive perfectionists to tasks were notably longer compared with those of the other groups (54). While it became clearly apparent in the sports tasks that the increased presence of perfectionist concerns accompanied with negative feedback has performance-degrading effects, adaptive perfectionists may more likely display slower reaction times due to their preference for precision and accuracy, at the expense of time.

### 4.4 Classification by dependent variables related to biomarkers

Numerous studies have demonstrated a correlation between perfectionism and physiological stress responses, particularly concerning the effect of negative feedback. High-level adequacy to social expectations (SPP) combined with a low self-confidence resulted in a significant increase in heart rate and showed a significant correlation with systolic blood pressure increase in response to negative feedback alone, whereas the impacts were slighter or absent in response to positive feedback (36). In contrast to other studies, perfectionists did not show significantly lower heart rate variability under stress compared with either the control group or their baseline values, indicating different patterns of physiological adaptation to stress (55). In another study of patients diagnosed with anorexia nervosa, the dorsal anterior cingulate cortex (dACC) activation was augmented in response to negative feedback, showing significant positive correlation with perfectionism, whereas this relationship was not observed in the control group (56). Furthermore, adaptive perfectionists reacted to negative feedback with significantly higher feedback-related negativity (FRN) amplitudes than in maladaptive perfectionists, indicating different patterns of neurocognitive processing (54). Such findings suggest that negative feedback may evoke more detrimental physiological outcomes as it preferentially activates threat-related neurobiological systems. Beyond perfectionism itself, co-occurring mechanisms—such as a heightened sensitivity to social evaluation, intolerance of uncertainty, or generally elevated threat-appraisal tendencies—can similarly amplify autonomic and neural stress responses. These pathways may help explain why negative feedback consistently produces stronger biomarker reactivity across studies, even when the perfectionism levels vary.

Of the 24 included studies, half of the interventions used a two-way instruction, therefore accompanied by both negative and positive feedbacks in these experiments.

The included literature also covered three studies representing different interventions as they applied an educational model, a specific form of assessment, or a personalized feedback method designed for perfectionists instead of bipolar positive or negative feedback. Relevant studies evaluated the impacts of these approaches.

One of the interventions, which implemented the MooN program (a physical education program), revealed a small effect but significant difference for self-reported perfectionism, although the mean value of the Perfectionism Scale did not show significant differences in the experimental group compared with the control group (57). Another impact study on exerting the influence of perfectionism among students learning English as a foreign language found no significant differences between the application of the interactionist dynamic assessment, the interventionist dynamic assessment, and the traditional education method (58). Finally, a third study, employing a method developed specifically for perfectionists, showed significantly increased self-affirmation, coping self-efficacy, and problem-focused coping in addition to decreased depressive and anxiety symptoms as a result of the intervention among perfectionists in the intervention group compared with the control group (59). In terms of the positive outcomes on perfectionism, there have been no studies in public education, but interventions among students in higher education have been confirmed successful. The sample sizes of these studies were similar; however, not only the sex ratio but also the age range of the participants differed. Thus, the results may have been potentially influenced by maturity of personality. Nevertheless, the methodology was the main difference between studies as only one intervention can be identified as specifically designed to address the issue of perfectionism.

### 4.5 Limitation analysis

Analysis of the articles was conducted based on four categories of limitations: the valence of feedback, the nature of the tasks performed by participants, the questionnaires used to assess perfectionism, and the generalizability of the findings to the sample populations. Information explicitly reported by the authors in the results sections of the articles was automatically included in the limitations summary table. In cases where no clear statement with regard to limitations or study weaknesses was provided, the decision to enter a “yes” or a “no” value in the respective field was made based on (60) consensus among the authors. As all of the included studies employed intervention-based methodologies, the sample sizes were generally small, and such small samples further limit the statistical power and robustness of the reported effects. While a few studies conducted prior sample size calculations and designed their research accordingly, this approach was not typical across the majority of the studies. During the screening process, only English-language publications were included; however, these originated from both Eastern and Western cultures, allowing for a degree of international generalizability. It is important to note, however, that 17 of the 24 studies utilized samples composed of university students, which limits the generalizability of the findings primarily to this population. This limitation is further compounded by the fact that university student samples are relatively homogeneous in demographic and psychological characteristics and differ in several respects from the general population (61). Prior methodological analyses have demonstrated that such samples possess specific cognitive and motivational profiles, which may introduce population bias and reduce external validity (62). Moreover, the manifestation of perfectionism may vary across ages, genders, and cultural backgrounds (63–65). Accordingly, the mechanisms identified in the present review are primarily applicable to student populations. Further research is needed to determine the extent to which these processes appear in different age groups, cultural contexts, or professional populations. The majority of the analyzed studies, with a few exceptions, investigated the complexity of the perfectionism construct using validated questionnaires. However, the included studies employed a variety of validated perfectionism measures that differ in their underlying theoretical emphases and assessed dimensions. This measurement heterogeneity limits the direct comparability of the intervention outcomes and therefore warrants cautious interpretation of the synthesized findings. In line with this, the review relied on narrative rather than quantitative synthesis as the variability in the outcome measures and the methodological approaches did not allow for a meaningful aggregation or a meta-analytic calculation of effect sizes. As a result, the magnitude of the reported associations could not be synthesized quantitatively; however, the narrative patterns identified across studies still allowed for meaningful interpretation of the relationship between feedback and perfectionism. A formal risk of bias assessment was not conducted, which represents a methodological limitation of the review. In addition, the possibility of publication bias cannot be ruled out as studies with null or non-significant findings may be underrepresented in the available literature. In conclusion, the predominance of positive values in the limitation table (**Table 2**) suggests that the mechanism identified in the experimental studies between perfectionism and feedback systems can be considered generalizable—primarily to university student populations.

**Table 2:** Study limitations.

No.	Study	The intervention includes both positive and negative feedback valence	The task appears to be both psychometrically and ecologically valid	The questionnaire provides a comprehensive overview of the construct of perfectionism	The results appear to be generalizable beyond the sample
1	Hobden and Pliner (1995) (48)	No	Yes	Yes	Yes
2	Besser et al. (2004) (41)	Yes	No	Yes	Yes
3	Anshel and Mansuori (2005) (50)	No	Yes	Yes	No
4	Besser et al. (2008) (36)	Yes	No	Yes	Yes
5	Stoeber et al. (2008) (39)	Yes	Yes	No	Yes
6	Kobori et al. (2009) (45)	No	Yes	Yes	No
7	Diamond et al. (2012) (46)	Yes	Yes	Yes	No
8	Egan et al. (2012) (47)	Yes	Yes	Yes	No
9	Azam et al. (2015) (55)	Yes	No	Yes	Yes
10	Cotet and Veresezan (2015) (44)	No	Yes	Yes	No
11	Muralidharan et al. (2015) (42)	No	Yes	No	No
12	Geisler et al. (2017) (56)	Yes	Yes	Yes	No
13	Curran and Hill (2018) (43)	No	No	Yes	Yes
14	Lo and Abbott (2019) (37)	Yes	Yes	Yes	Yes
15	Cooks and Ciesla (2019) (38)	Yes	Yes	Yes	Yes
16	Lizmore et al. (2019) (51)	No	No	Yes	No
17	Luna et al. (2021) (57)	Not relevant	Yes	Yes	Yes
18	Rahmaty and Zarei (2021) (58)	Not relevant	Yes	Yes	Yes
19	De Muynck et al. (2021) (40)	Yes	No	No	No
20	Isheqlou et al. (2022) (53)	Yes	Yes	Yes	Yes
21	Dunkley et al. (2023) (59)	Not relevant	Yes	Yes	No
22	Isheqlou et al. (2023) (54)	Yes	Yes	Yes	Yes
23	Portešová et al. (2023) (49)	Not relevant	No	Yes	No
24	Zhou et al. (2025) (52)	No	No	Yes	Yes

### 4.6 Framing theoretical insights

**Figure 2:**
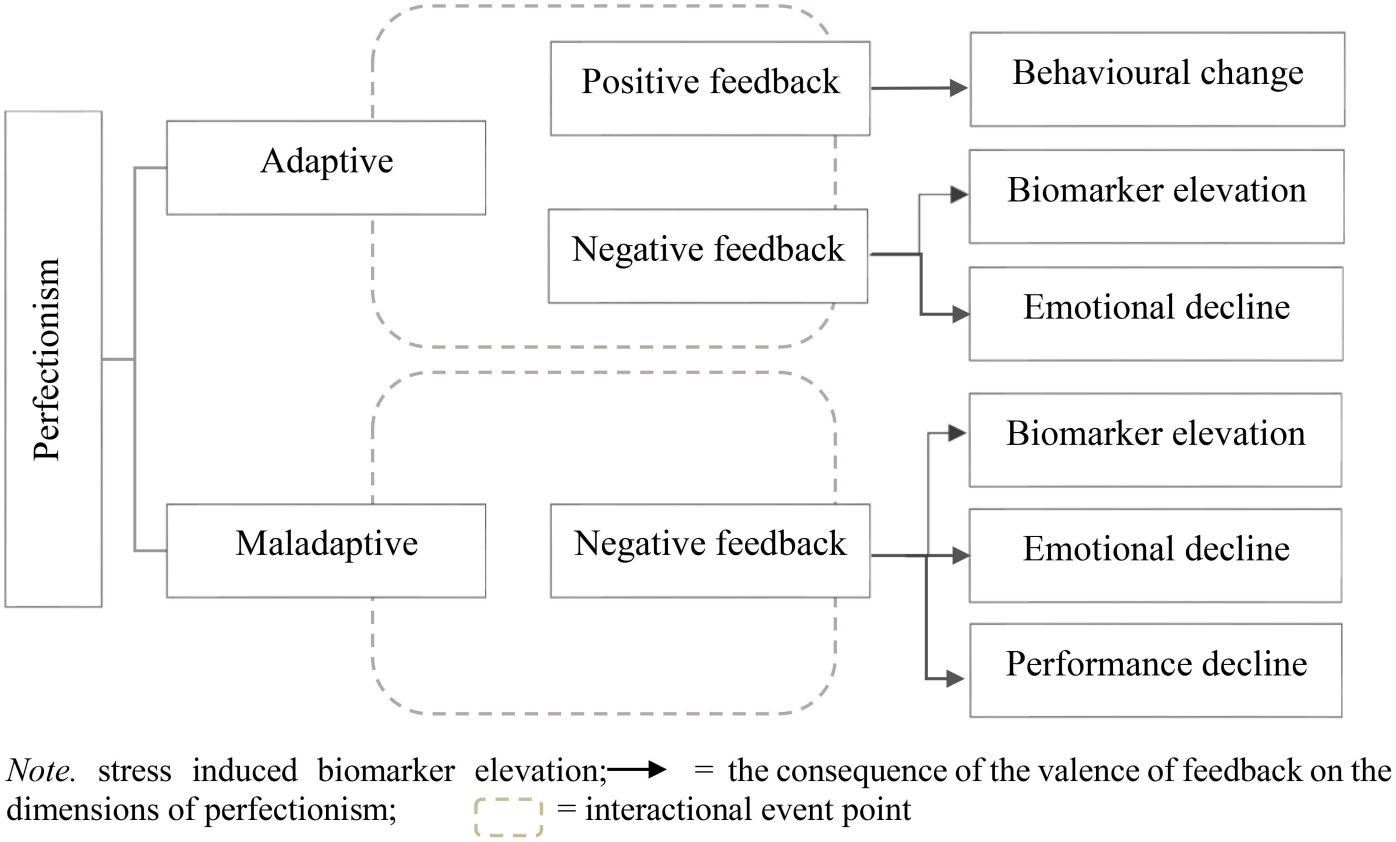
Interaction model of the perfectionism dimensions and feedback valence.

The Bibliometric–Systematic Literature Review (B-SLR) approach builds on the integration of the PRISMA protocol with other established tools and frameworks to address the disconnections between bibliometric analyses, SLRs, and theory development (66). In alignment with the 10th step of the B-SLR—i.e., developing a theoretical contribution—a corresponding theoretical framework was developed. The theoretical model constructed on the basis of the results and our conclusions, illustrating the mechanism by which positive and negative feedbacks affect the dimensions of perfectionism, is presented in **Figure 2**.

A theoretical grounding for the model is provided by two influential frameworks on perfectionism and feedback processing. Firstly, the multidimensional model of perfectionism proposed by Frost et al. (20) highlights the heightened emotional vulnerability characteristic of maladaptive dimensions—particularly the concern over mistakes and doubts about actions. This perspective helps explain why negative feedback produces a more consistent and a pronounced cognitive and emotional deterioration among individuals high in maladaptive perfectionism. Secondly, the dual process model developed by Slade and Owens (67), which is rooted in reinforcement theory, which posits that the adaptive and maladaptive forms of perfectionism are maintained by distinct motivational processes: adaptive perfectionism is more responsive to positive reinforcement, whereas maladaptive perfectionism is sustained primarily through negative reinforcement cycles. This framework aligns closely with the patterns reflected in our model, namely, that the consequences of negative feedback are more stable and detrimental, whereas the effects of positive feedback are less predictable and not uniformly beneficial.

Psychological and physiological consequences are evident in both the adaptive and maladaptive forms of perfectionism in response to feedback. However, the performance decline appears to be associated exclusively with the maladaptive perfectionism when exposed to negative feedback. In contrast, adaptive perfectionism in the context of positive feedback may lead to behavioral changes in multiple directions, potentially encompassing both positive and negative shifts.

The model does not reflect a symmetrical impact of feedback valence across the two perfectionism subtypes. Specifically, positive feedback is not linked to maladaptive perfectionism, as we concluded during the theoretical model development process that no stable or consistent outcomes can be derived from such an association. Overall, the consequences depicted in the theoretical model are predominantly negative in nature: this is reflected in the stress-induced biomarker reactivity, the emotional deterioration, and the decline in performance.

## 5 Conclusion

During the systematic literature review, we did not find studies that address the development of perfectionism. Thus, the actual underlying mechanisms behind the issue remain unclear beyond the previous theoretical framework. However, the results confirm a clear link between feedback and the perfectionism dimensions. Negative feedback leads to emotional and cognitive deterioration in both the adaptive and maladaptive forms, with a stronger effect on the latter. Positive feedback can produce improvements, but these are infrequent and not generalizable. Behavioral findings support this pattern. Overall, negative feedback has a detrimental effect on the performance-related outcomes and induces stronger physiological stress responses, as reflected in the biomarker changes. In conclusion, future research should apply multiple perfectionism questionnaires within a single experimental design and include both positive and negative feedback conditions to better understand their distinct effects. Based on the findings of the present systematic review, future research would benefit from employing more complex study designs in which the key components represented in the interaction model of perfectionism dimensions and feedback valence—i.e., behavioral, emotional, performance-related, and biomarker indices—are assessed within a single experimental framework. Such a multimodal approach would substantially enhance the precision with which the mechanisms linking the perfectionism dimensions to feedback valence can be identified. Methodological consistency may be further strengthened through the adoption of more standardized reporting practices, including *a priori* sample size planning and a detailed documentation of the feedback procedures. Establishing such a comprehensive research framework could contribute to a more refined understanding of the multifaceted nature of perfectionism and aid in distinguishing which observed effects are genuinely attributable to perfectionism, as opposed to other less well-controlled factors.

Despite existing therapies, developmental interventions rarely reach educational, sports, or workplace settings, leaving many emotional, behavioral, and performance-related issues unaddressed. Although therapeutic methods promote supportive environments, the current findings suggest that negative feedback has more pronounced psychological and physiological impacts on perfectionists than positive input.

## Data Availability

The original contributions presented in the study are included in the article/supplementary material. Further inquiries can be directed to the corresponding author.
